# Supplementary figures and images for: Evaluation of RNAi and CRISPR technologies by large-scale gene expression profiling in the Connectivity Map
Source: PLoS Biol. 2017 Nov 30;15(11):e2003213. doi: 10.1371/journal.pbio.2003213 (PMC5726721; doi:10.1371/journal.pbio.2003213)

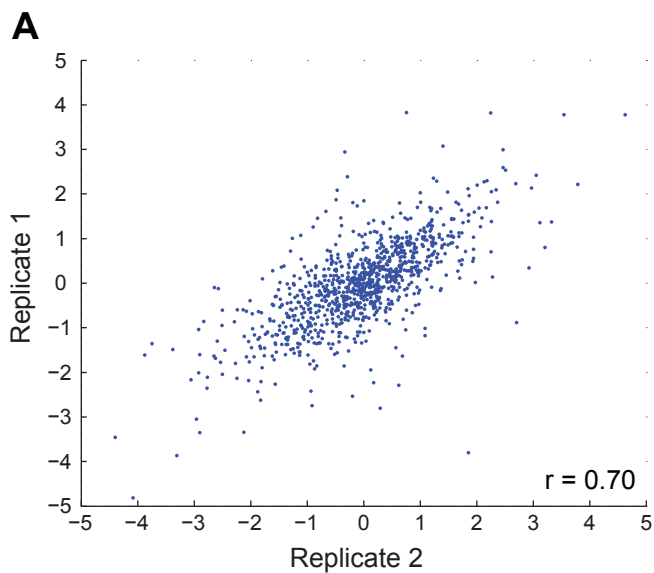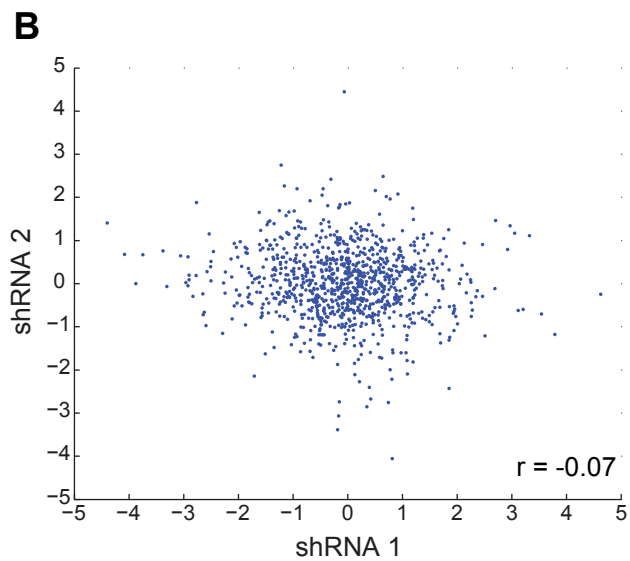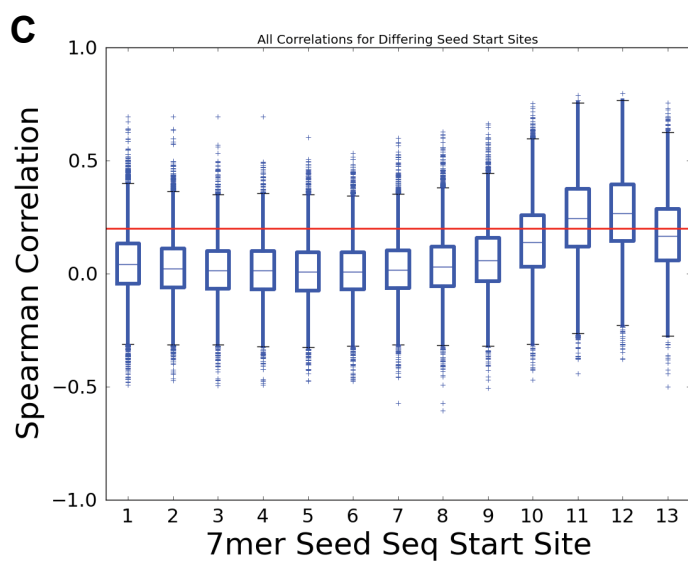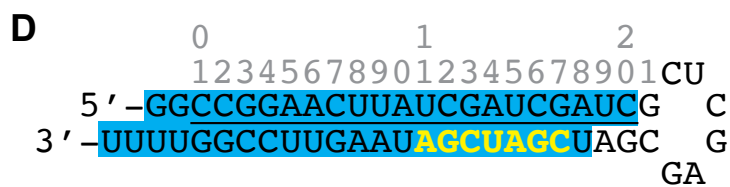

Supplement: S1 Fig — (A) An example of strong correlation: 2 replicates of the same control shRNA show strong reproducibility as measured by the Spearman correlation of the differential expression of the 978 measured landmark genes. Each point represents 1 landmark transcript. (B) An example of no correlation between signatures of different shRNAs. (C) 7-mers beginning at positions 11 and 12 of the annotated sense strand show the greatest correlation, corresponding to the seed sequence of the antisense/targeting strand, and reflecting heterogeneity of Dicer processing. See S1 Data. (D) Schematic of shRNAs used in CMAP. The 21-nt sense strand, which has the same sequence as the target mRNA, is underlined and numbered. The blue highlight indicates the major siRNA product produced after Dicer processing. The bolded, yellow nts indicate the seed sequence of the antisense/targeting strand. CMAP, Connectivity Map; nt, nucleotide; shRNA, short hairpin RNA; siRNA, small interfering RNA. (PDF) [file pbio.2003213.s001.pdf]

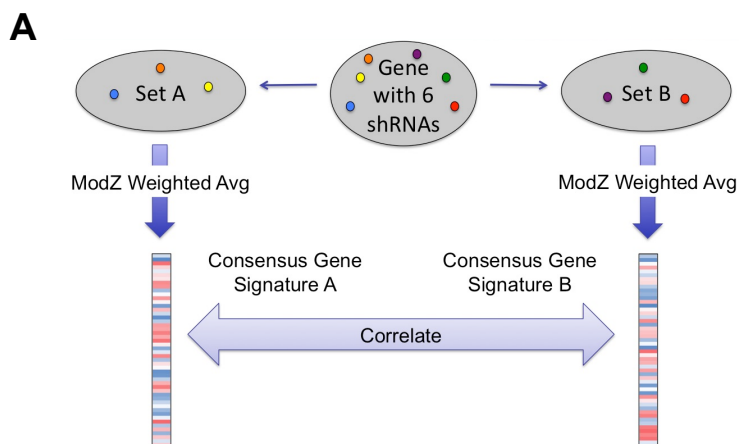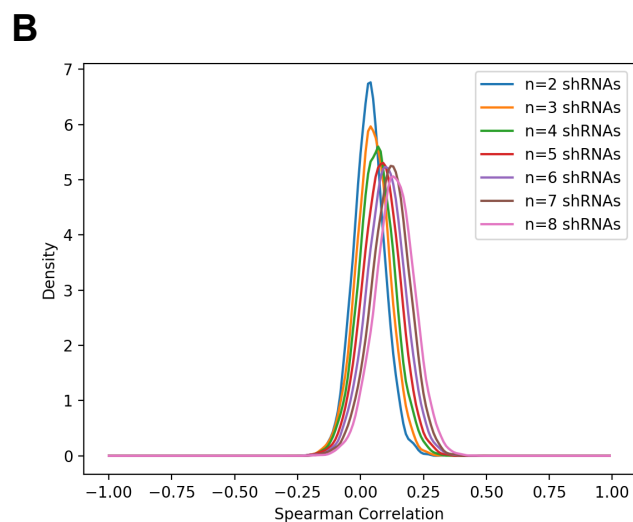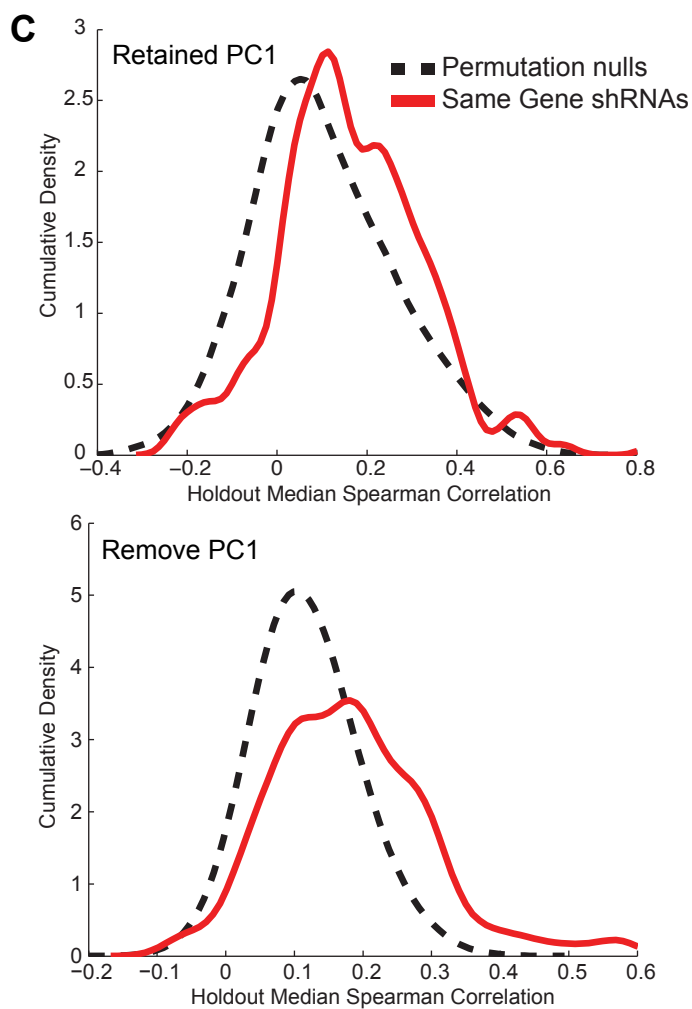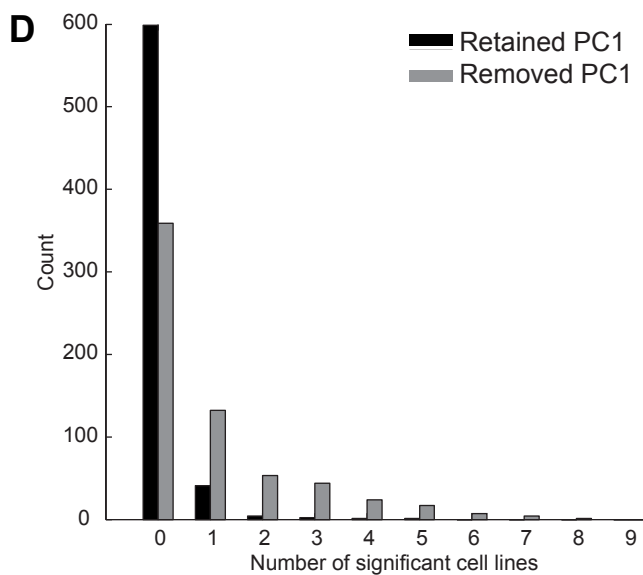

Supplement: S2 Fig — (A) Schematic of holdout analysis. For genes with 6 or more shRNAs, the CGS is calculated from subsets of shRNAs, and the resulting CGSs are correlated. This procedure is repeated with different random partitions of shRNAs. (B) The distributions for null signatures, comprising random draws of shRNAs, with PC1 removed. Compare to Fig 2B. (C) Comparison of holdout correlations to permutations nulls in an example cell line. Top: unmodified signatures; Bottom: PC1 removed. Removing PC1 increases the fraction of genes with a statistically significant correlation. (D) For each gene, the number of cell lines in which the CGS passes statistical significance for holdout analysis for unmodified data (black) and for data with the PC1 removed (gray). See S4 Data. CGS, consensus gene signature; shRNA, short hairpin RNA; PC1, first principal component. (PDF) [file pbio.2003213.s002.pdf]

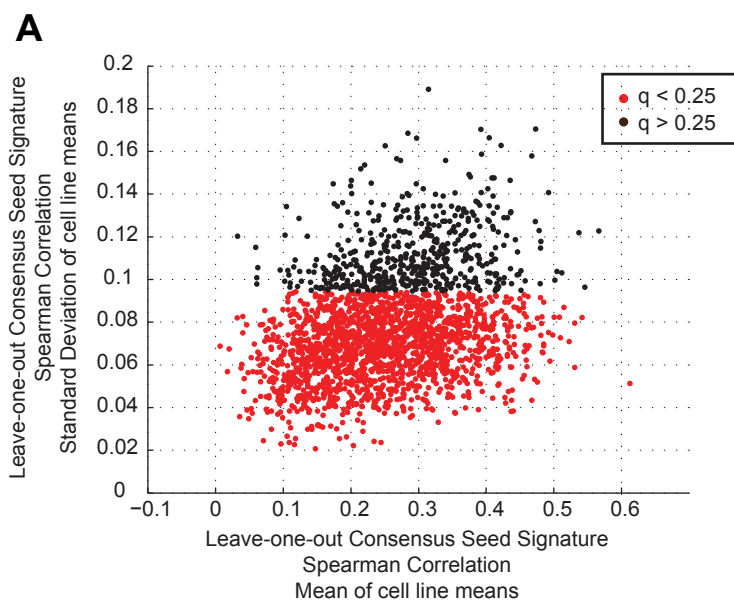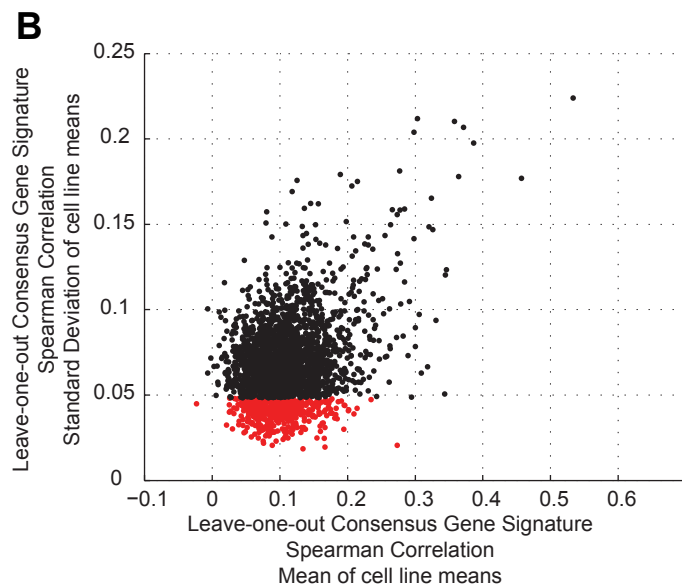

Supplement: S3 Fig — For each seed sequence (A) or gene target (B), the mean correlation to the leave-one-out CSS or CGS is calculated within each cell line. The vector of means by cell line for each seed or gene can be compared to the collection of means by cell line for all seeds or genes. Seeds and genes that cause gene expression changes of the same magnitude (not necessarily direction) will tend to have smaller variance than the population, reflecting that consistency. These scatter plots show the mean (x-axis) and standard deviation (y-axis) of the vector of means across the 9 cell lines. Those in red are different than the population per the F-test at an FDR of <25%. Note that the threshold for significance is different for genes and seeds because genes and seeds have different leave-one-out correlation distributions. CGS, consensus gene signature; CSS, consensus seed signature; FDR, false discovery rate. (PDF) [file pbio.2003213.s003.pdf]

Replicate correlation vs roast projection RMS magnitude, LINCS core hairpins, n=115750  
Pearson Corr = 0.7834

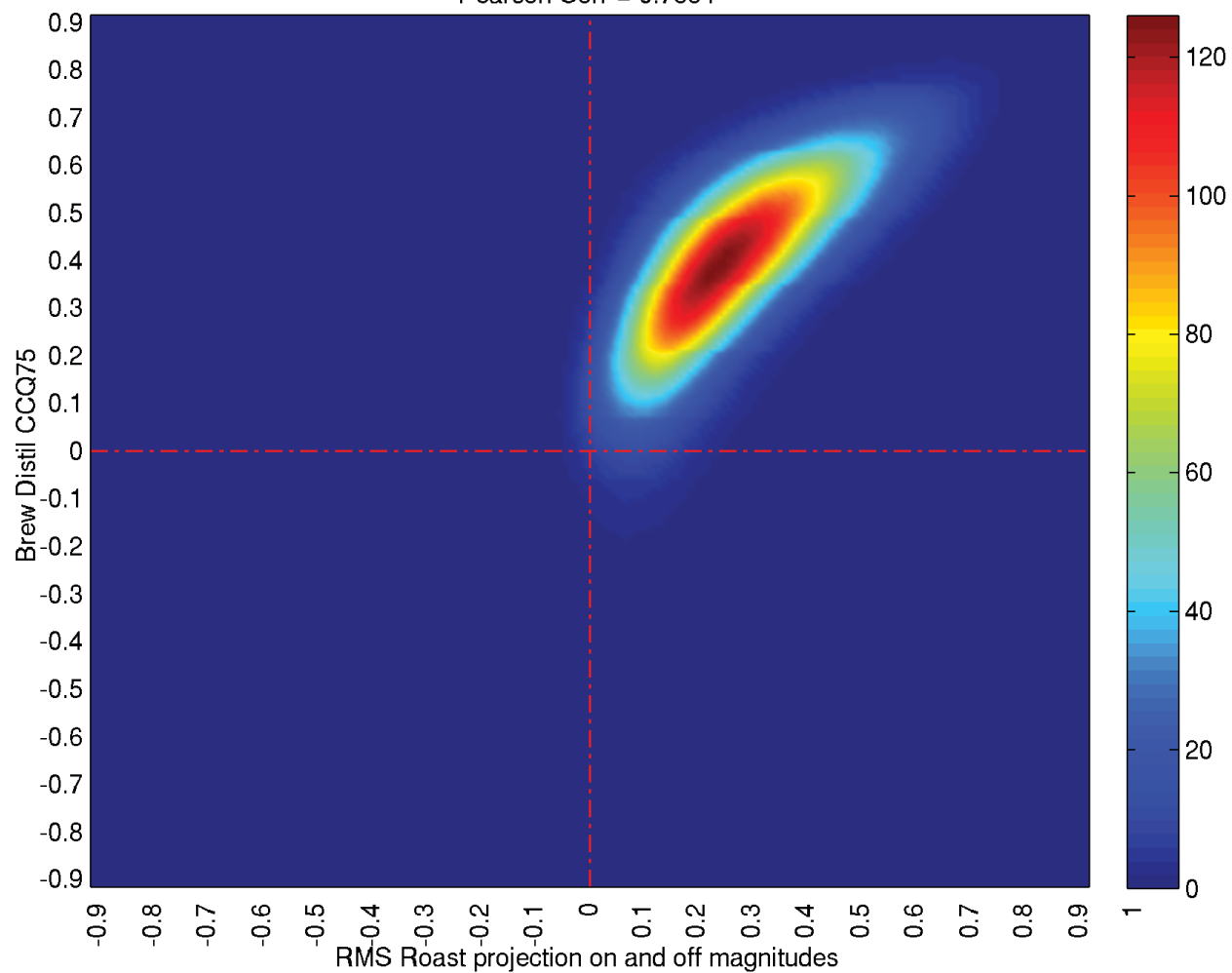

Supplement: S4 Fig — The projection algorithm decomposes any particular signature into on-target and residual relative magnitudes. These represent the entirety of the reproducible part of the signature; the remainder is noise. The Pythagorean theorem gives the length of that reproducible part as the square root of the sum of the squares of the 2 projection magnitudes. This projection length correlates very well with the independently measured replicate correlation. For the set of all shRNAs, the x-axis is the calculated projection length, and the y-axis is the 75th quantile of Spearman correlation among replicates. The 2 quantities correlate with a Pearson correlation of 0.78, providing a sanity check that the projection outputs are meaningful. The nonlinearity is due to the use of the 75th quantile for replicate correlation by convention, compared to the use of the mean for projection length. shRNA, short hairpin RNA. (PDF) [file pbio.2003213.s004.pdf]
